# Doctor-patient bilateral matching considering diagnosis and treatment perception in the absence of public health resources

**DOI:** 10.3389/fpubh.2022.1094523

**Published:** 2023-01-19

**Authors:** Wangqi Zhu, Jitao He, Hangxin Guo

**Affiliations:** Huaxin Consulting Co., Ltd., Hangzhou, China

**Keywords:** doctor-patient bilateral matching, diagnosis and treatment perception, public health, multi-objective decision-making, NSGA-II algorithm

## Abstract

**Introduction:**

The public health crisis is one of the main threats affecting the sustainable development of the economy and strengthening the rational allocation of medical resources is essential for building a strong public health system. Therefore, the study of the doctor-patient bilateral matching has important theoretical and practical significance and perception of diagnosis and treatment is taken as a key consideration in the research.

**Methods:**

Based on the current situation of the medical industry and the main contradiction between supply and demand of medical services, an evaluation index of doctor-patient satisfaction is constructed in this paper. Then, based on the different forms of evaluation, calculate the doctor's satisfaction and patient's satisfaction respectively. Taking maximizing the overall satisfaction of doctors and patients, maximizing the number of patients and minimizing the workload difference between doctors as the decision-making objectives, considering the upper limit of doctors' working hours as the constraint condition, a multi-objective decision-making model is constructed and solved by NSGA-II algorithm to realize the matching between doctors and patients.

**Conclusion:**

Finally, through the comparison with NSGA-III algorithm in three dimensions: the degree of convergence to the reference set, the propagation range of the solution and the running time of the algorithm, it is proved that NSGA-II algorithm has good performance in solving the matching problem of medical service supply and demand.

## 1. Introduction

People's health is the most basic livelihood issue and a strong public health system provides a strong guarantee for people's health. The improvement of people's material living standards has fundamentally changed people's understanding of medical and health values (1). As a world populous country, China has 18% of the world's population, but public health resources are relatively scarce. At the same time, today's medical service is expanding to “prevention-treatment-rehabilitation-health care” based on the traditional treatment of diseases. Under the condition of limited medical resources, this will inevitably lead to the phenomenon of “medical congestion”. The phenomenon of “medical congestion”, that is, the limited medical resources can't meet the growing needs of patients, is essentially an imbalance between doctors and patients. In hospitals with serious “medical congestion”, doctors have a greater probability of overload, which will affect the professionalism of doctors in the process of diagnosis and treatment to a certain extent. Research shows that about 70% of urban hospitals will turn ambulances due to “medical congestion”, resulting in some emergency patients missing the golden age of treatment (2). In addition, “medical congestion” is easy to prolong patients' waiting time for treatment, treatment cycle and treatment cost, resulting in low satisfaction and becoming the fuse of doctor-patient contradictions (3). Therefore, applying advanced information technology and products to the daily practice of medical management, integrating existing resources and realizing the reasonable matching between doctors and patients on the premise of ensuring the continuous increase of medical investment, has very important practical significance for improving the operation efficiency of medical work, optimizing the allocation of medical resources and promoting the development of public health (4).

Academia has applied the bilateral matching theory to many different fields. Liu et al. have applied the bilateral matching theory to the field of education to improve the perceived satisfaction of teachers and students through the matching decision between graduate freshmen and tutors, and solved the decision model with genetic algorithm (5). Wu et al. applied the bilateral matching theory to the financial field to solve the matching problem between financial products and the actual needs of enterprises from the perspective of enterprise risk bearing and financing theory (6). Jiang and Yuan applied the bilateral matching theory to the field of human resources to realize the bilateral matching between the existing post holders and external applicants by considering the fairness of competition and the stability of employees (7). Zhu et al. applied the field of bilateral matching to the field of transportation, and studied the matching between vehicle source and goods source by considering the fairness and satisfaction of matching subjects (8). Cao and Yang applied the bilateral matching theory to the communication field and studied the uplink NOMA user pairing method (9). Yu applied the bilateral matching theory to the practice of PPP projects and studied the impact of different matching mechanisms on the game results (10).

Some scholars have also applied the bilateral matching theory to the practice of medical management, but there are few studies on the matching between doctors and patients. Considering the coexistence of patients' expectation hesitation and determination in the diagnosis and treatment process, Lu et al. constructed a multi-objective function with the goal of maximizing the comprehensive satisfaction of the matching subject and minimizing the difference between patients and doctors. When solving, the multi-objective function was transformed into a single objective function for solution to realize the accurate matching of doctors and patients in the context of remote treatment (11). Wang et al. used the two-stage matching method to divide the matching between patients and doctors into two stages. Firstly, patients are divided into balanced groups according to the individual needs of patients. Then, the patient satisfaction is calculated according to the patient's expectations, and the matching between patients and doctors is realized by establishing a matching model (12). When building the decision-making model, Chen and Wang considered the hesitation and uncertainty of doctors and patients, and studied the matching between doctors and patients on the intelligent diagnosis and treatment platform by calculating the difference between the expectation and the actual situation of both sides (13). Zhong introduced the matching theory into the practical application of the medical and health industry and proposed that doctors' personalized preference plays a vital role in the stability of team work. Therefore, he considered the personalized preference of patients in the research process (14). Gao et al. considered the individual needs of patients and took maximizing doctor-patient satisfaction as the decision-making goal to realize doctor-patient bilateral matching (15). From the perspective of patients' needs, Yuan et al. considered the attention to the differences of doctors' attributes and doctors' operation types in the process of diagnosis and treatment, and studied the matching strategy taking the satisfaction and stability of doctors and patients into account (16). Ferreira et al. established a multi-objective decision-making model to match the satisfaction of both parties and maximize the utilization efficiency of medical resources, and studied the surgical resource scheduling problem (17). Neyshabouri and Berg proposed a two-stage decision-making matching method to realize the matching between patients and medical resources in consideration of the differences in the skills required by patients (18).

To sum up, the existing research provides a reference for doctor-patient bilateral matching, but there are also some deficiencies. Although the psychological perception of both matching parties is considered in literature (19), the multi-objective function is transformed into a single objective function when solving, which will lead to the complex topology of the weighted objective function and the deviation between the decision result and the actual result. Literature (12, 14) only consider the satisfaction of one of the matching subjects, which may reduce the patient's experience and the doctor's work efficiency. Literature (13, 15, 16) consider the satisfaction of doctors and patients, but does not take the measurement of workload among doctors into account, which may lead to overload of some doctors. Therefore, this paper takes maximizing the overall satisfaction of doctors and patients, maximizing the number of patients and minimizing the workload difference between doctors as the decision-making objectives, considers the upper limit of doctors' working hours as the constraint, constructs a multi-objective decision-making model, and proposes a matching method that fully considers the actual demands of doctors and patients in the diagnosis and treatment process.

## 2. Problem description and symbol description

### 2.1. Problem description

The essence of doctor-patient bilateral matching decision is to form the doctor-patient matching result with the highest comprehensive utility by using technical means or management methods based on certain decision-making objectives. When patients receive medical services, they have psychological expectations for the doctors who provide medical services to themselves, usually including professional technology, moral cultivation, charging standard and so on. When patients register on the reservation platform, they can express their needs on the platform and form a patient expectation matrix. Similarly, doctors also have psychological expectations for the objects they provide medical services, mainly including the expectations of patients' disease types and patients' quality. Doctors can submit their preferences to hospital management decision-makers to form a doctor expectation matrix.

**Figure 1:**
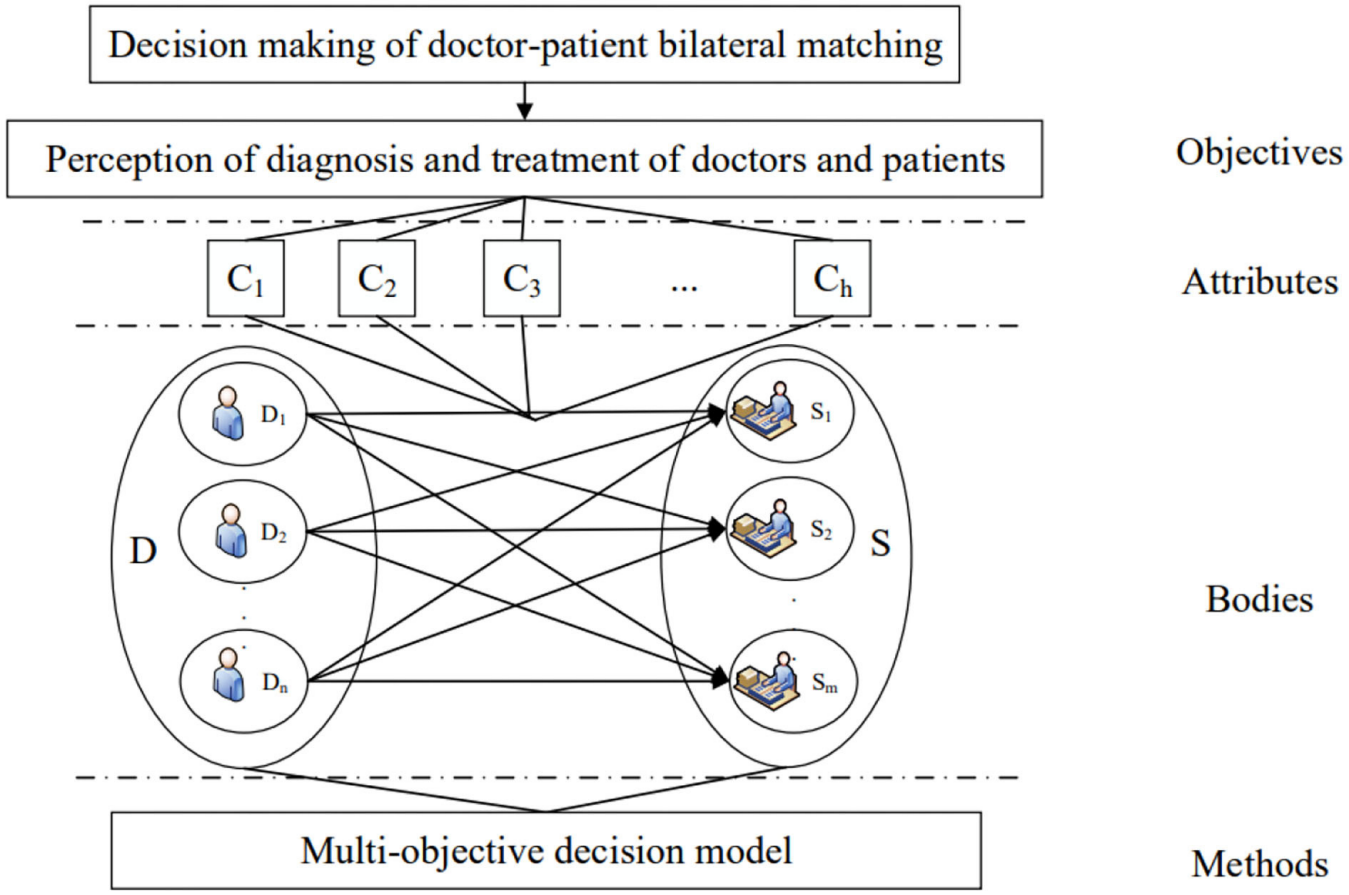
The hierarchical structure of doctor-patient bilateral matching decision.

At the same time, hospital management decision-makers need to objectively evaluate doctors and patients based on the same dimension to calculate doctors' satisfaction and patients' satisfaction. In the decision-making process, the decision-makers aim to meet the expectations of both doctors and patients as much as possible, take the number of patients and doctors' workload into account, and comprehensively weigh among various factors to form a matching pair with the highest comprehensive utility. The hierarchical structure of doctor-patient bilateral matching decision is shown in Figure 1.

### 2.2. Symbol description

(*D*_1_, *D*_2_, …, *D*_*n*_): Collection of patients, where *D*_*i*_ represents patient *i*, *i* = 1, 2, …, *n*.

(*S*_1_, *S*_2_, …, *S*_*k*_): Collection of doctors, where *S*_*m*_ represents patient *m*, *m* = 1, 2, …, *k*.

(*C*_1_, *C*_2_, …, *C*_*h*_) : The collection of attributes of the patient's evaluation index to the doctor, where *C*_*l*_ represents attribute *l*, *l* = 1, 2, …, *h*. Matching attributes have three forms: clarity number, interval number and language evaluation.

(*Q*_1_, *Q*_2_, …, *Q*_*j*_): The collection of the attributes of the doctor's evaluation indicators for patients, where *Q*_υ_ represents attribute υ, υ = 1, 2, …, *j*. The form of matching attribute is the same as above.

*E* = [*e*_*il*_]_*n*×*h*_: The expectation matrix of the patient to the doctor, where *e*_*ig*_ represents the expectation level of the patient *D*_*i*_ about the attribute *C*_*l*_ of the medical service, *i* = 1, 2, …, *n*, *l* = 1, 2, …, *h*.

*F* = [*e*_*mα*_]_*k*×*j*_: The expectation matrix of the doctor to the patient, where *e*_*mα*_ represents the expectation level of the doctor *S*_*m*_ about the attribute *Q*_α_ of the medical service, *m* = 1, 2, …, *k*, α = 1, 2, …, *j*.

*A* = [*a*_*ml*_]_*k*×*h*_: The evaluation matrix of the decision-maker to the doctor, where *a*_*ml*_ represents the evaluation level of the decision-maker on the attribute *C*_*l*_ of doctor *S*_*m*_, *m* = 1, 2, …, *k*, *l* = 1, 2, …, *h*.

*B* = [*a*_*iα*_]_*n*×*j*_: The evaluation matrix of the decision-maker to the patient, where *a*_*iα*_ represents the evaluation level of the decision-maker on the attribute *Q*_α_ of patient *D*_*i*_, *i* = 1, 2, …, *n*, α = 1, 2, …, *j*.

*p*_*i*_: Severity of patient *D*_*i*_. It is divided into five levels of 1–5, of which the larger the number, the more serious the patient's condition is.

*t*_*i*_: Estimated treatment time of patient *D*_*i*_.

*T*: The maximum working hours per doctor per day.

## 3. Model construction of doctor-patient bilateral matching decision

### 3.1. Construction of evaluation index system for doctor satisfaction and patient satisfaction

Based on the current main contradiction between supply and demand of medical services, the evaluation index system of doctor-patient satisfaction is constructed. The construction process of the index system follows the principles of professionalism, comprehensiveness and timeliness. Professionalism: Research on the evaluation index system of academic doctor-patient satisfaction, combined with the visit and investigation of front-line medical workers, to ensure the professionalism of the index system from two aspects of professional theory and reality; Comprehensiveness: fully consider patients' expectations on doctors' professional skills, moral cultivation, charging standards, and doctors' expectations on patients' condition and quality, and ensure that the evaluation indicators fully reflect the psychological feelings of the matching subjects in the diagnosis and treatment process; Timeliness: considering the characteristics of the times of the medical industry, for example, today's medicine has changed from a simple biomedical model to a combination of “biomedicine + social psychology”, so social psychological factors should be fully considered when constructing indicators.

Therefore, patients comprehensively evaluate whether doctors meet their expectations from five aspects professional level (*C*_1_), service attitude (*C*_2_), reputation (*C*_3_), humanistic concerns (*C*_4_), and fees (*C*_5_). At the same time, doctors' preferences for patients are mainly considered from the four dimensions of resource urgency (*Q*_1_), expertise similarity (*Q*_2_), cooperation (*Q*_3_), and patience (*Q*_4_). Among them, cost indicators include fees (*C*_5_) and resource urgency (*Q*_1_), and the rest are benefit indicators.

### 3.2. Calculate patient satisfaction

Patients put forward their expectations for doctors from different dimensions and decision-makers evaluate doctors from the same dimension, and then consider whether the actual situation of doctors meets the needs of patients. Therefore, to measure this satisfaction, it is necessary to calculate the patient satisfaction based on the patient's expectation matrix and the doctor's evaluation matrix. Patients' evaluation of doctors mainly includes clarity number, interval number and language evaluation. Specifically, the satisfaction calculation formula of the three evaluation forms is as follows.

#### 3.2.1. Patient satisfaction when the evaluation type is clear number

Suppose that the patient's expectation level for the doctor is *e*_*il*_ = ${e_{il}}^{\prime }$, and the decision-maker's evaluation level for the doctor is *a*_*ml*_ = ${a_{ml}}^{\prime }$, where ${e_{il}}^{\prime }$ ≥ 0, ${a_{ml}}^{\prime }$ ≥ 0. At this time, for attribute *C*_*l*_, the satisfaction $u_{im}^{l}$ of patient *D*_*i*_ to doctor *S*_*m*_ is calculated as follows:

When the index is a benefit index:

(1) $$\begin{array}{l} u_{im}^{l}\\ =\{\begin{array}{l} \frac{e_{il}^{{\;}^{\prime }}-a_{ml}^{{\;}^{\prime }}}{e_{il}^{{\;}^{\prime }}},e_{il}^{{\;}^{\prime }}>a_{ml}^{{\;}^{\prime }};\\ 1\;,e_{il}^{{\;}^{\prime }}\leq a_{ml}^{{\;}^{\prime }}. \end{array}i=1,2,...,n,m=1,2,...,k,l=1,2,...,h \end{array}$$

When the indicator is a cost indicator:

(2) $$\begin{array}{l} u_{im}^{l}\\ =\{\begin{array}{l} \;1\;,e_{il}^{{\;}^{\prime }}\geq a_{ml}^{{\;}^{\prime }};\\ \frac{a_{ml}^{{\;}^{\prime }}-e_{il}^{{\;}^{\prime }}}{e_{il}^{{\;}^{\prime }}},e_{il}^{{\;}^{\prime }}<a_{ml}^{{\;}^{\prime }}. \end{array}i=1,2,...,n,\;m=1,2,...,k,\;l=1,2,...,h \end{array}$$

Where benefit indicators are positive indicators (i.e., the larger the better), and cost indicators are negative indicators (i.e., the smaller the better).

#### 3.2.2. Patient satisfaction when the evaluation type is interval number

Suppose that the patient's expectation level for the doctor is *e*_*il*_ = $\left[e_{il}^{L},e_{il}^{R}\right]$, and the decision-maker's evaluation level for the doctor is *a*_*ml*_, where $e_{il}^{R}$ ≥ $e_{il}^{L}$ ≥ 0. At this time, for attribute *C*_*l*_, the satisfaction $u_{im}^{l}$ of patient *D*_*i*_ to doctor *S*_*m*_ is calculated as follows:


(3) $$\begin{array}{l} u_{im}^{l}\\ =\{\begin{array}{l} \frac{e_{il}^{L}-a_{ml}}{e_{il}^{L}},a_{ml}<e_{il}^{L};\\ 1,e_{il}^{L}\leq a_{ml}\leq e_{il}^{R};\\ \frac{a_{ml}-e_{il}^{R}}{e_{il}^{R}},a_{ml}>e_{il}^{R}. \end{array}i=1,2,...,n,\;m=1,2,...,k,\;l=1,2,...,h \end{array}$$

#### 3.2.3. Patient satisfaction when the evaluation type is short sentences

Suppose that the patient's short sentence evaluation of the doctor is represented by *O* = {*O*_1_, …, *O*_*g*_}, where *O*_*c*_ represents the short sentence *c* in set *O*, and *g* is the granularity of set *O*. Suppose that the granularity level of the patient's expectation level *e*_*il*_ of the doctor is expressed as δ_*il*_ (e.g., when the patient's evaluation of the doctor is *O*_3_, δ_*il*_ = 3), and the granularity level of the decision-maker's evaluation level *a*_*ml*_ of the doctor is expressed as β_*ml*_. At this time, for attribute *C*_*l*_, the satisfaction $u_{im}^{l}$ of patient *D*_*i*_ to doctor *S*_*m*_ is calculated as follows:

When the index is a benefit index:


(4) $$\begin{array}{l} u_{im}^{l}\\ =\{\begin{array}{l} \frac{\delta_{il}-\beta_{ml}}{\delta_{il}},\delta_{il}>\beta_{ml};\\ 1\;,\delta_{il}\leq \beta_{ml}. \end{array}i=1,2,...,n,m=1,2,...,k,\;l=1,2,...,h \end{array}$$

When the indicator is a cost indicator:

(5) $$\begin{array}{l} u_{im}^{l}\\ =\{\begin{array}{l} \;1\;,\delta_{il}\geq \beta_{ml};\\ \frac{\beta_{ml}-\delta_{il}}{\beta_{ml}},\delta_{il}<\beta_{ml}. \end{array}i=1,2,...,n,\;m=1,2,...,k,\;l=1,2,...,h \end{array}$$

Based on the above different types of evaluation forms, calculate patient satisfaction $u_{im}^{l}$. Combined with weight *w*_*l*_, the comprehensive satisfaction of patient *D*_*i*_ to doctor *S*_*m*_ is:

(6) $$\begin{array}{l} U_{im}=\overset{h}{\underset{l=1}{\sum }}w_{l}u_{im}^{l} \end{array}$$

### 3.3. Calculate doctor satisfaction

Like patients, doctors also have psychological expectations for patients in the process of diagnosis and treatment. Based on the expectation matrix of doctors for patients, decision-makers evaluate patients from the same dimension, and then consider whether the actual situation of patients meets the needs of doctors.

#### 3.3.1. Doctor satisfaction when the evaluation type is clear number

Suppose that the doctor's expectation level for the patient is *e*_*mυ*_ = ${e_{m\upsilon }}^{\prime }$, and the decision-maker's evaluation level for the patient is *a*_*iυ*_ = ${a_{i\upsilon }}^{\prime }$, where ${e_{m\upsilon }}^{\prime }$ ≥ 0, ${a_{i\upsilon }}^{\prime }$ ≥ 0. At this time, for attribute *Q*_υ_, the satisfaction $u_{mi}^{\upsilon }$ of doctor *S*_*m*_ to patient *D*_*i*_ is calculated as follows:

(7) $$\begin{array}{l} u_{mi}^{\upsilon }=\{\begin{array}{l} \frac{{e_{m\upsilon }}^{\prime }-{a_{i\upsilon }}^{\prime }}{{e_{m\upsilon }}^{\prime }},\;{e_{m\upsilon }}^{\prime }>{a_{i\upsilon }}^{\prime };\\ 1\text{,}{e_{m\upsilon }}^{\prime }\leq {a_{i\upsilon }}^{\prime }. \end{array}\;i=1,2,...,n,\;m=1,2,...,k,\;\\ \;\upsilon =1,\text{2,}...\text{,}j \end{array}$$

#### 3.3.2. Doctor satisfaction when the evaluation type is short sentences

The doctor's short sentences evaluation of the patient can refer to the form of the patient's short sentences of the doctor, expressed as *O* = {*O*_1_, …, *O*_*g*_}, where *O*_*c*_ represents the short sentence *c* in set *O*, and *g* is the granularity of set *O*. Suppose that the granularity level of the doctor's expectation level *e*_*mυ*_ of the patient is expressed as γ_*mυ*_, and the granularity level of the decision-maker's evaluation level *a*_*iυ*_ of the doctor is expressed as η_*iυ*_. At this time, for attribute *Q*_υ_, the satisfaction $u_{mi}^{\upsilon }$ of doctor *S*_*m*_ to patient *D*_*i*_ is calculated as follows:

(8) $$\begin{array}{l} u_{mi}^{\upsilon }=\{\begin{array}{l} \frac{\eta_{i\upsilon }-\gamma_{m\upsilon }}{\eta_{i\upsilon }},\;\eta_{i\upsilon }>\gamma_{m\upsilon };\\ 1\text{,}\gamma_{m\upsilon }\leq \eta_{i\upsilon }. \end{array}\;i=1,2,...,n,m=1,2,..,k,\upsilon =1,\text{2,}..\text{,}j \end{array}$$

Based on the above different types of evaluation forms, calculate doctor satisfaction $u_{im}^{\upsilon }$. Combined with weight *w*_χ_, the comprehensive satisfaction of doctor *S*_*m*_ to patient *D*_*i*_ is:

(9) $$\begin{array}{l} {\overset{-}{U}}_{mi}=\overset{j}{\underset{\upsilon =1}{\sum }}w_{\chi }u_{mi}^{\upsilon } \end{array}$$

### 3.4. Construction of multi-objective decision model

Suppose that the matching variable *x*_*im*_ between the doctor and the patient follows the 0–1 integer programming, that is, when the matching between patient *D*_*i*_ and doctor *S*_*m*_ is successful, *x*_*im*_ = 1; Conversely, if there is no match, *x*_*im*_ = 0.

In the process of establishing the decision-making model, while meeting the basic diagnosis and treatment needs of patients, further consider the negative impact of the workload on doctors and diagnosis and treatment results, and measure it from the following three factors:

Factor 1: The difference in the number of patients received by each doctor should be as small as possible.

(10) $$\begin{array}{l} \min\overset{q-1}{\underset{m=1}{\sum }}\overset{q}{\underset{r>m}{\sum }}|\overset{n}{\underset{i=1}{\sum }}x_{im}-\overset{n}{\underset{i=1}{\sum }}x_{ir}| \end{array}$$

Factor 2: Each doctor should accept the number of patients with the same severity as possible.

(11) $$\begin{array}{l} \min\overset{q-1}{\underset{m=1}{\sum }}\overset{q}{\underset{r>m}{\sum }}|\overset{n}{\underset{i=1}{\sum }}p_{i}x_{im}-\overset{n}{\underset{i=1}{\sum }}p_{i}x_{ir}| \end{array}$$

Factor 3: The difference in the working hours of each doctor every day should be as small as possible.

(12) $$\begin{array}{l} \min\overset{q-1}{\underset{m=1}{\sum }}\overset{q}{\underset{r>k}{\sum }}|\overset{n}{\underset{i=1}{\sum }}t_{i}x_{im}-\overset{n}{\underset{i=1}{\sum }}t_{i}x_{ir}| \end{array}$$

Suppose *v*_1_, *v*_2_ and *v*_3_ are the weight of three factors that balance the workload between doctors. A multi-objective decision-making model for doctor-patient bilateral matching is constructed based on three factors: satisfaction, number of patients and doctor workload.

(13) $$\begin{array}{l} \{\begin{array}{l} \max Z_{1}=\overset{n}{\underset{i=1}{\sum }}\overset{k}{\underset{m=1}{\sum }}U_{im}x_{im}+\overset{n}{\underset{i=1}{\sum }}\overset{k}{\underset{m=1}{\sum }}{\overset{-}{U}}_{mi}x_{im}\\ \max Z_{2}=\overset{n}{\underset{i=1}{\sum }}\overset{k}{\underset{m=1}{\sum }}x_{im}\\ \min Z_{3}=v_{1}\overset{q-1}{\underset{m=1}{\sum }}\overset{q}{\underset{r>m}{\sum }}|\overset{n}{\underset{i=1}{\sum }}x_{im}-\overset{n}{\underset{i=1}{\sum }}x_{ir}|\\ +v_{2}\overset{q-1}{\underset{m=1}{\sum }}\overset{q}{\underset{r>m}{\sum }}|\overset{n}{\underset{i=1}{\sum }}p_{i}x_{im}-\overset{n}{\underset{i=1}{\sum }}p_{i}x_{ir}|\\ +v_{3}\overset{q-1}{\underset{m=1}{\sum }}\overset{q}{\underset{r>k}{\sum }}|\overset{n}{\underset{i=1}{\sum }}t_{i}x_{im}-\overset{n}{\underset{i=1}{\sum }}t_{i}x_{ir}|\\ s.t.\overset{k}{\underset{m=1}{\sum }}x_{im}\leq 1\\ s.t.\overset{n}{\underset{i=1}{\sum }}t_{i}x_{im}\leq T \end{array} \end{array}$$

Where max *Z*_1_ means to maximize the comprehensive satisfaction of doctors and patients in the decision-making process; max *Z*_2_ means to maximize the number of patients who can receive treatment; max *Z*_3_ means to make a decision to balance the workload among doctors as much as possible; $\overset{k}{\underset{m=1}{\sum }}x_{im}\leq 1$ means that each patient can only be matched with one doctor at most; $\overset{n}{\underset{i=1}{\sum }}t_{i}x_{im}\leq T$ represents the maximum working hours of each doctor in a single day.

## 4. Solution method of doctor-patient bilateral matching decision model

Due to the slow speed of NSGA algorithm in solving large-scale problems, as well as the restriction of objective conditions such as manually specifying the sharing radius and no elite selection strategy, it cannot reflect the good performance of the solution. Based on this, Srinivas et al. improved NSGA algorithm and proposed NSGA-II algorithm. NSGA-II algorithm further reduces the computational complexity and improves the performance in solving multi-objective decision-making problems through three steps of fast non-dominated sorting, crowding comparison operator and elite retention strategy, on the basis of ensuring population diversity (19).

The main process steps of NSGA-II algorithm are as follows:

Step 1: Generate the initial population with population size of *N*, denoted as *P*_*n*_, and the evolution algebra of initialization population *n* = 0;

Step 2: The initial population *P*_*n*_ is sorted into different levels according to the difference of dominance degree, and the crowding distance of each individual is calculated at the same time;

Step 3: Select, cross and mutate the parent population to form the child population *Q*_*n*_, and combine the child population with the parent population to form a new population set, which is recorded as *P*_2*N*_.

Step 4: For the new population set *P*_2*N*_, the elite retention strategy is adopted to retain individuals with high degree of non-domination and large crowding distance to form a new generation of population *P*_*n* + 1_.

Step 5: Repeat steps 2–4 until the population evolution algebra is greater than the set population evolution algebra.

## 5. Analog simulation

In order to make full use of medical resources and improve operation efficiency, the decision-makers of oral hospital hope to form a good match between doctors and patients through the reform of management system, so as to balance the workload between doctors while improving the psychological feelings of patients and doctors in the process of diagnosis and treatment.

### 5.1. Bilateral matching decision of doctor-patient based on NSGA-II algorithm

There were 18 patients with dental pulp disease, which was denoted as set *D* = {*D*_1_, *D*_2_, *D*_3_, …, *D*_18_}, and the patient's characteristic information is shown in Table 1.

**Table 1:** Characteristic information of patients.

**Patient number**	**Age**	**Sex**	**Illness degree**	**Estimated time of diagnosis (h)**	**Patient number**	**Age**	**Sex**	**Illness degree**	**Estimated time of diagnosis (h)**
*D*_1_	31	M	2	1.5	*D*_10_	46	M	3	2.5
*D*_2_	37	M	3	2	*D*_11_	39	M	4	1.5
*D*_3_	48	F	2	1.5	*D*_12_	33	F	5	3
*D*_4_	21	F	2	1	*D*_13_	34	M	2	1.5
*D*_5_	26	M	4	2	*D*_14_	33	M	3	2
*D*_6_	23	F	1	0.5	*D*_15_	20	F	4	2.5
*D*_7_	18	F	3	2	*D*_16_	54	M	3	2
*D*_8_	29	M	4	2.5	*D*_17_	52	F	2	1
*D*_9_	53	F	2	0.5	*D*_18_	47	M	2	1

There are five doctors in oral hospital who treat dental pulp diseases, and the collection of doctors is *S* = {*S*_1_, *S*_2_, …, *S*_5_}. The expectations of patients for dentist are shown in Table 2.

**Table 2:** Patients' expectations of doctors.

	***C*_1_**	***C*_2_**	***C*_3_**	***C*_4_**	***C*_5_**		***C*_1_**	***C*_2_**	***C*_3_**	***C*_4_**	***C*_5_**
*D*_1_	9	*O*_4_	*O*_3_	*O*_3_	[80, 100]	*D*_10_	9	*O*_3_	*O*_3_	*O*_3_	[80, 120]
*D*_2_	9	*O*_4_	*O*_5_	*O*_4_	[75, 120]	*D*_11_	8	*O*_3_	*O*_4_	*O*_4_	[80, 100]
*D*_3_	8	*O*_3_	*O*_4_	*O*_2_	[60, 90]	*D*_12_	9	*O*_4_	*O*_3_	*O*_3_	[80, 100]
*D*_4_	10	*O*_5_	*O*_3_	*O*_4_	[70, 100]	*D*_13_	7	*O*_5_	*O*_4_	*O*_3_	[65, 95]
*D*_5_	8	*O*_4_	*O*_3_	*O*_3_	[90, 140]	*D*_14_	9	*O*_3_	*O*_3_	*O*_4_	[60, 80]
*D*_6_	7	*O*_3_	*O*_3_	*O*_3_	[50, 90]	*D*_15_	9	*O*_4_	*O*_3_	*O*_5_	[70, 105]
*D*_7_	8	*O*_3_	*O*_3_	*O*_2_	[70, 105]	*D*_16_	9	*O*_4_	*O*_4_	*O*_4_	[75, 115]
*D*_8_	10	*O*_5_	*O*_4_	*O*_4_	[95, 140]	*D*_17_	8	*O*_5_	*O*_4_	*O*_4_	[60, 75]
*D*_9_	8	*O*_4_	*O*_3_	*O*_3_	[65, 100]	*D*_18_	7	*O*_4_	*O*_4_	*O*_3_	[55, 80]

Among the five evaluation dimensions, *C*_1_ is the evaluation dimension in the form of clear number, expressed as an integer between 0 and 10, of which 10 points represent the most satisfied and 0 points represent the least satisfied; *C*_2_, *C*_3_ and *C*_4_ are the evaluation dimensions expressed in the form of language short sentences, which are divided into five levels according to the degree of evaluation, namely *O* = {*O*_1_ = very poor, *O*_2_ = poor, *O*_3_= medium, *O*_4_ = good, *O*_5_ = very good}; *C*_5_ is the evaluation dimension expressed in the form of interval number, which means that the satisfaction of patients in this interval is maximized. Based on these five evaluation dimensions, patients put forward their expectations for doctors and get the expectation matrix of patients, as shown in Table 2. At the same time, hospital decision-makers will also objectively evaluate doctors based on these five evaluation dimensions and the evaluation level of doctors is shown in Table 3.

**Table 3:** Assessment level of doctors.

	***C*_1_**	***C*_2_**	***C*_3_**	***C*_4_**	***C*_5_**
*S*_1_	7	*O*_5_	*O*_3_	*O*_4_	72
*S*_2_	8	*O*_4_	*O*_3_	*O*_4_	78
*S*_3_	9	*O*_3_	*O*_3_	*O*_3_	90
*S*_4_	10	*O*_4_	*O*_5_	*O*_4_	110
*S*_5_	9	*O*_3_	*O*_4_	*O*_3_	88

In the doctor's evaluation dimension of patients, *Q*_1_ and *Q*_2_ are the evaluation dimensions in the form of clear numbers, *Q*_3_ and *Q*_4_ are the evaluation dimensions in the form of short sentences. Based on these four evaluation dimensions, doctors put forward their expectations for patients and get the doctor's expectation matrix, as shown in Table 4. At the same time, hospital decision-makers will also objectively evaluate patients based on these four evaluation dimensions and the evaluation level of patients is shown in Table 5.

**Table 4:** Doctors' expectations for patients.

	***Q*_1_**	***Q*_2_**	***Q*_3_**	***Q*_4_**
*S*_1_	8	9	*O*_3_	*O*_4_
*S*_2_	7	8	*O*_3_	*O*_4_
*S*_3_	9	9	*O*_4_	*O*_3_
*S*_4_	9	7	*O*_5_	*O*_5_
*S*_5_	9	8	*O*_4_	*O*_4_

**Table 5:** Hospital evaluation level of patients.

**Patients**	***Q*_1_**	***Q*_2_**	***Q*_3_**	***Q*_4_**	**Patients**	***Q*_1_**	***Q*_2_**	***Q*_3_**	***Q*_4_**
*D*_1_	8	9	*O*_5_	*O*_5_	*D*_10_	9	8	*O*_3_	*O*_3_
*D*_2_	9	8	*O*_5_	*O*_3_	*D*_11_	8	8	*O*_5_	*O*_4_
*D*_3_	8	9	*O*_4_	*O*_2_	*D*_12_	10	10	*O*_5_	*O*_4_
*D*_4_	7	8	*O*_4_	*O*_4_	*D*_13_	7	9	*O*_4_	*O*_3_
*D*_5_	8	10	*O*_3_	*O*_3_	*D*_14_	8	8	*O*_3_	*O*_3_
*D*_6_	7	8	*O*_4_	*O*_3_	*D*_15_	9	9	*O*_4_	*O*_5_
*D*_7_	8	9	*O*_3_	*O*_2_	*D*_16_	7	7	*O*_4_	*O*_4_
*D*_8_	10	10	*O*_4_	*O*_5_	*D*_17_	7	8	*O*_5_	*O*_4_
*D*_9_	6	7	*O*_3_	*O*_3_	*D*_18_	7	8	*O*_4_	*O*_3_

**Figure 2:**
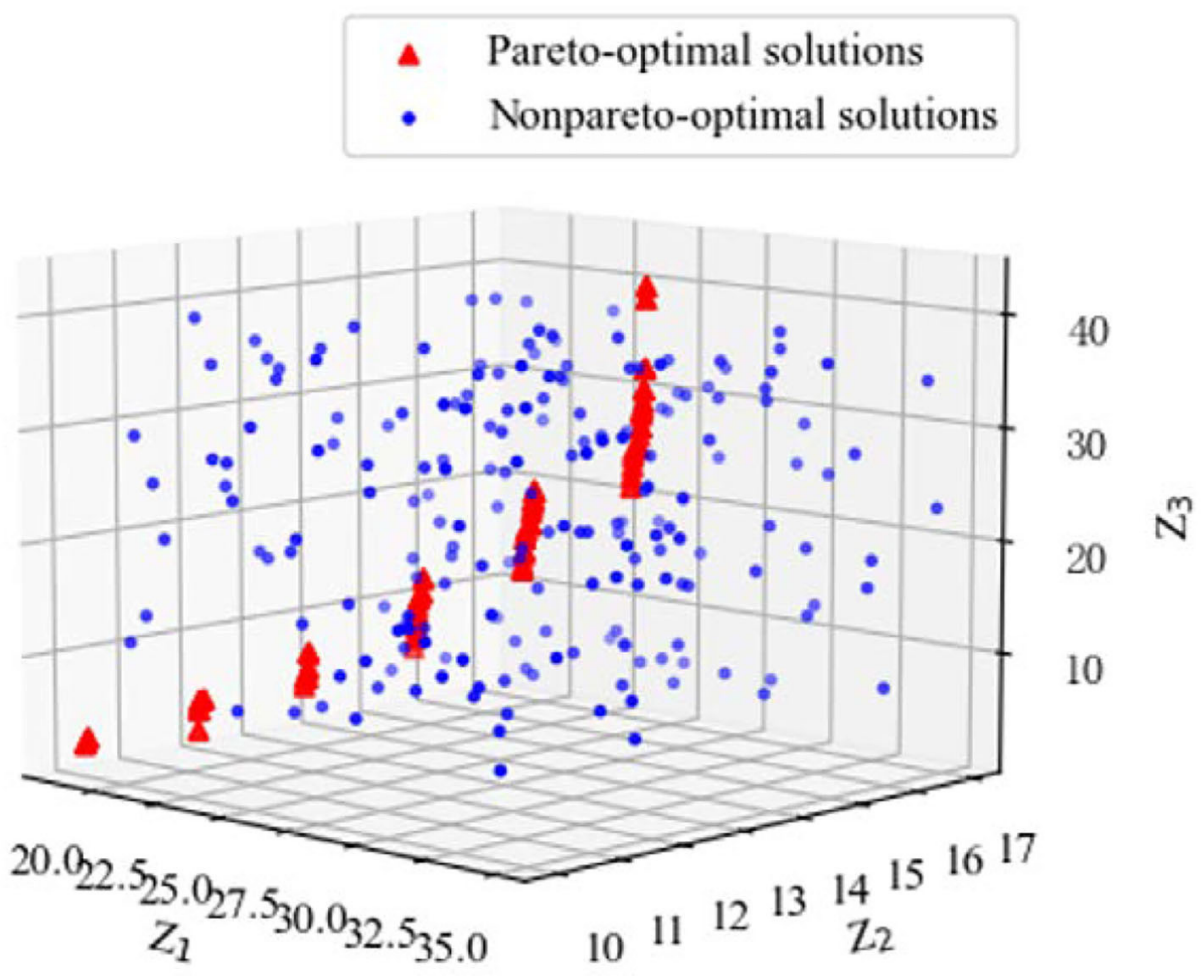
Feasible solution of doctor-patient bilateral matching decision model.

In the decision-making process, the influence of doctors' working hours on diagnosis and treatment focus and treatment effect is fully considered, so each dentist is set to work no more than 10 h a day, that is, *T* = 10. Doctor satisfaction and patient satisfaction can be calculated through Formula (1)–(9), and a target model based on doctor-patient satisfaction can be constructed. At the same time, considering the maximization of patient reception and the minimization of workload difference between doctors, the weights of the three decision objectives are determined as ν_1_ = 0.35, ν_2_ = 0.28, and ν_3_ = 0.37, respectively, by Delphi method, based on which the doctor-patient bilateral matching decision model is constructed. NSGA-II algorithm is used to solve the model, and the population size *n* = 200, genetic algebra *gen* = 200, crossover probability*pc* = 0.95, mutation probability *pm* = 0.05 are set. The Pareto optimal solution is shown in Table 6, and the feasible solution is shown in Figure 2. The red triangle constitutes the solution plane satisfying the Pareto optimal condition.

**Table 6:** Pareto optimal solutions.

	**Pareto optimal solution (target space)** $X={\left(x_{1,1},\;x_{1,2},x_{1,3},x_{1,4},x_{1,5},......,x_{18,1},x_{18,2},x_{18,3},x_{18,4},x_{18,5}\right)}^{T}$	**Optimal value**		
		*Z*_1_	*Z*_2_	*Z*_3_
1	*x*_1,3_ = 1, *x*_2,2_ = 1, *x*_3,1_ = 1, *x*_4,5_ = 1, *x*_6,3_ = 1, *x*_7,1_ = 1, *x*_8,1_ = 1, *x*_9,4_ = 1, *x*_10,2_ = 1, *x*_11,5_ = 1, *x*_13,1_ = 1, *x*_14,1_ = 1, *x*_16,4_ = 1, *x*_17,2_ = 1, *x*_18,3_ = 1.	27.8	15	24.8
2	*x*_1,3_ = 1, *x*_2,1_ = 1, *x*_3,1_ = 1, *x*_4,5_ = 1, *x*_6,3_ = 1, *x*_7,1_ = 1, *x*_9,4_ = 1, *x*_10,2_ = 1, *x*_11,5_ = 1, *x*_13,1_ = 1, *x*_14,2_ = 1, *x*_15,1_ = 1, *x*_16,4_ = 1, *x*_17,2_ = 1, *x*_18,3_ = 1.	27.8	15	27.0
3	*x*_1,3_ = 1, *x*_4,5_ = 1, *x*_5,1_ = 1, *x*_6,3_ = 1, *x*_7,1_ = 1, *x*_8,1_ = 1, *x*_9,2_ = 1, *x*_10,2_ = 1, *x*_11,5_ = 1, *x*_12,4_ = 1, *x*_13,1_ = 1, *x*_14,2_ = 1, *x*_15,1_ = 1, *x*_17,2_ = 1, *x*_18,3_ = 1.	28.3	15	34.7
4	*x*_1,3_ = 1, *x*_2,1_ = 1, *x*_3,1_ = 1, *x*_4,5_ = 1, *x*_5,1_ = 1, *x*_6,3_ = 1, *x*_8,1_ = 1, *x*_9,4_ = 1, *x*_10,2_ = 1, *x*_11,5_ = 1, *x*_13,1_ = 1, *x*_14,2_ = 1, *x*_16,4_ = 1, *x*_17,2_ = 1, *x*_18,3_ = 1.	27.8	15	25.1
5	*x*_1,3_ = 1, *x*_3,1_ = 1, *x*_4,5_ = 1, *x*_5,1_ = 1, *x*_6,3_ = 1, *x*_7,1_ = 1, *x*_8,1_ = 1, *x*_9,2_ = 1, *x*_10,2_ = 1, *x*_11,5_ = 1, *x*_12,4_ = 1, *x*_13,1_ = 1, *x*_14,2_ = 1, *x*_17,2_ = 1, *x*_18,3_ = 1.	28.2	15	31.0
6	*x*_1,3_ = 1, *x*_3,1_ = 1, *x*_4,5_ = 1, *x*_5,1_ = 1, *x*_6,3_ = 1, *x*_8,1_ = 1, *x*_9,2_ = 1, *x*_10,2_ = 1, *x*_11,5_ = 1, *x*_12,4_ = 1, *x*_13,1_ = 1, *x*_14,2_ = 1, *x*_15,1_ = 1, *x*_17,2_ = 1, *x*_18,3_ = 1.	28.2	15	32.8

### 5.2. Analysis of comparison results between NSGA-II algorithm and NSGA-III algorithm

#### 5.2.1. Comparative experimental method

To further verify the good performance of NSGA-II algorithm in solving large-scale problems, NSGA-II algorithm is compared with NSGA-III algorithm from three dimensions: convergence of algorithm solution, stability of solution distribution and algorithm running time:

(1) Convergence of solution τ. Firstly, the non inferior solutions obtained by NSGA-II algorithm and NSGA-III algorithm are combined to form a new solution set. Then calculate the average Euclidean distance from each feasible solution of the two algorithms to the nearest feasible solution in the new solution set. The formula can be expressed as:

(14) $$\begin{array}{l} \tau =\left(\overset{N}{\underset{i=1}{\sum }}d_{i}\right)/N \end{array}$$

Where *d*_*i*_ represents the average Euclidean distance between the feasible solution *i* and the nearest feasible solution in the new solution set, and *N* represents the total number of feasible solutions. The smaller the value of τ, the higher the degree of convergence of the solution.

(2) Stability of solution distribution ϖ. Firstly, the boundary solution of the feasible solution is determined, and then the Euclidean distance from the extreme solution to the boundary solution is calculated. The calculation formula can be expressed as:

(15) $$\begin{array}{l} \varpi =\frac{d_{1}+d_{2}+\sum_{i-1}^{N-1}|d_{i}-\overset{-}{d}|}{d_{1}+d_{2}+\left(N-1\right)\overset{-}{d}} \end{array}$$

Where *d*_1_ and *d*_2_ represent the Euclidean distance between the extreme solution and the boundary solution of the feasible solution, and $\bar{d}$ represents the average Euclidean distance. The smaller the value of ϖ, the better the stability of the solution distribution.

(3) Running time *t*. Record the running time of the single solution model of the algorithm.

The steps of the comparative experiment are as follows:

Step 1: Patient satisfaction matrix *M*_1_ = (*u*_*ik*_)_*n*×*m*_ and doctor satisfaction matrix *M*_2_ = (*u*_*ki*_)_*n*×*m*_ are randomly generated, respectively, where satisfaction follows the random distribution of [0,1].

Step 2: The patient's condition degree vector κ = (κ_1_, κ_2_, …, κ_*n*_) and the patient's estimated diagnosis and treatment time vector λ = (λ_1_, λ_2_, …, λ_*n*_) are randomly generated, in which the value of κ is randomly taken from set {1, 2, 3, 4, 5} and the value of λ is randomly taken from set {0.5, 1, 1.5, 2, 2.5, 3}. Based on this, the multi-objective decision-making model is constructed.

Step 3: The model is solved by NSGA-II algorithm and NSGA-III algorithm to obtain the running time of the algorithm. At the same time, the Pareto optimal solution sets *W*_1_ and *W*_2_ are obtained, respectively, and the set *W* = *W*_1_⋃*W*_2_.

Step 4: Calculate the values of τ and ϖ by Formulas (14) and (15).

#### 5.2.2. Comparative analysis of algorithms

By setting the number of patients *n*, the number of doctors *m* and the population size *N*, the problems of different sizes are constructed. For different situations, NSGA-II algorithm and NSGA-III algorithm are used to solve them for 10 times, respectively, and the average value and variance of τ and ϖ are calculated. The test sample parameters of the algorithm are shown in Table 7.

**Table 7:** Algorithm test sample.

	***n***	***m***	***N***	**Maximum genetic algebra**	***p*_c_**	***p*_m_**	**Solution space Ω**
1	20	5	100	100	0.95	0.05	*O* (20^5^)
2	30	7	100	100	0.95	0.05	*O* (30^7^)
3	40	9	150	100	0.95	0.05	*O* (40^9^)
4	50	10	200	100	0.95	0.05	*O* (50^10^)
5	60	12	200	100	0.95	0.05	*O* (60^12^)
6	70	15	200	100	0.95	0.05	*O* (70^15^)
7	80	18	300	200	0.95	0.05	*O* (80^18^)
8	100	20	300	200	0.95	0.05	*O* (100^20^)
9	110	22	300	200	0.95	0.05	*O* (110^22^)
10	120	25	500	200	0.95	0.05	*O* (120^25^)
11	130	28	500	200	0.95	0.05	*O* (130^28^)
12	140	30	500	200	0.95	0.05	*O* (140^30^)

**Figure 3:**
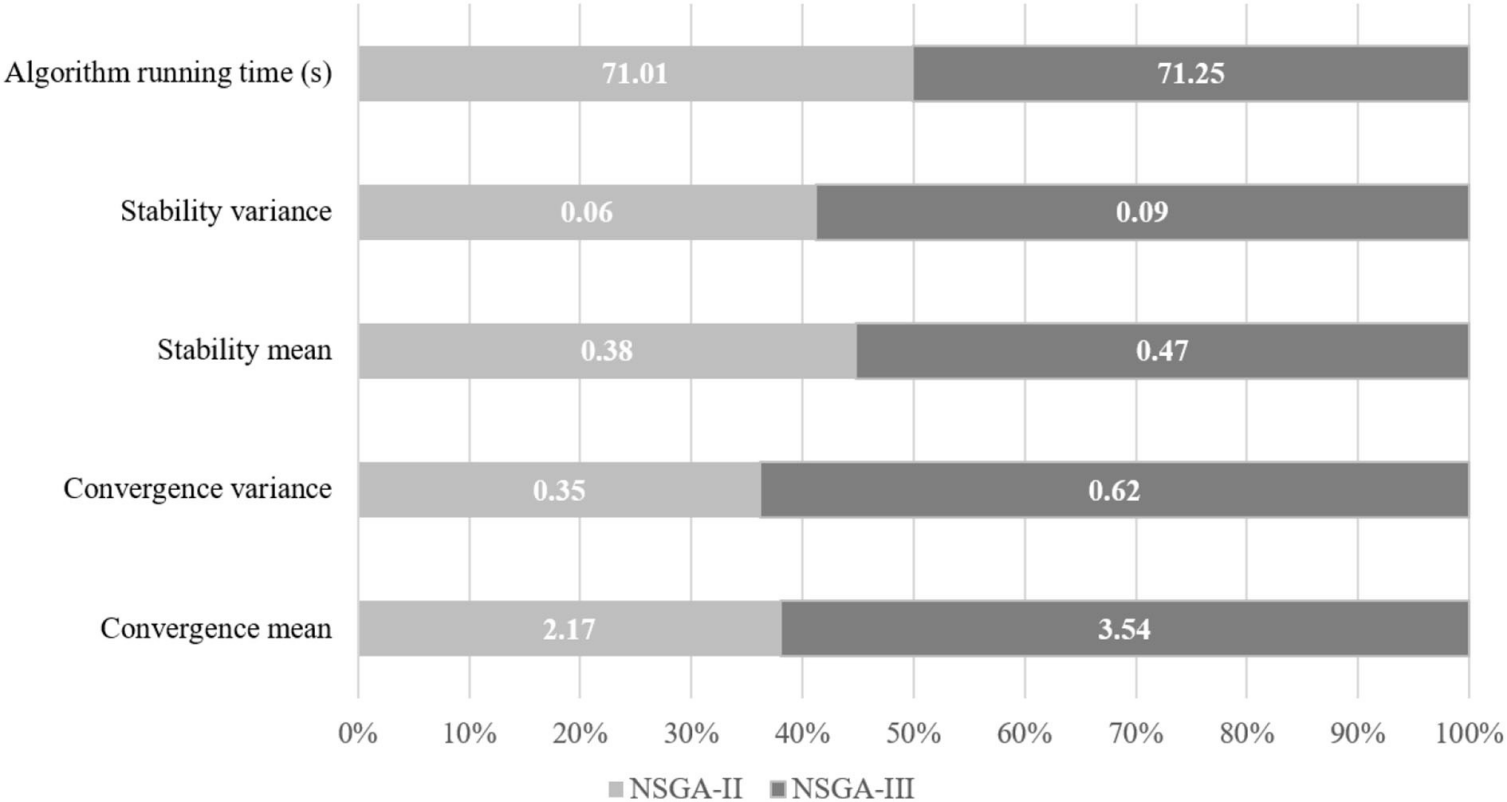
Performance comparison of NSGA-II and NSGA-III algorithms.

It can be seen from Figure 3 that from the two dimensions of solution convergence and stability of distribution, NSGA-II algorithm is generally smaller than NSGA-III algorithm in terms of mean and variance, which shows that NSGA-II algorithm is easier to produce non-inferior solutions close to the reference set and the generated non-inferior solutions have a more stable distribution. Therefore, compared with NSGA-III algorithm, NSGA-II algorithm has better convergence in solving large-scale problems, and the resulting non-inferior solutions have a more stable distribution. When solving large-scale problems, the running time of NSGA-II algorithm and NSGA-III algorithm in all test problems is very close, but the solution speed of NSGA-II algorithm is faster on the whole. It shows that NSGA-II algorithm produces non-inferior solutions faster in general.

Therefore, it can be concluded that NSGA-II algorithm has better performance in solving the problem of bilateral matching of doctor-patient.

## 6. Summary and prospect

The rational allocation of medical resources plays an important role in building a sound public health system. The outbreak of the COVID-19 epidemic has exposed the inadequacy of current public health planning. Studying how to realize the rational allocation of medical resources plays an important role in promoting the sustainable, stable and development of public health. This paper proposes a decision-making method of doctor-patient bilateral matching, which fully considers the actual demands of doctors and patients in the process of diagnosis and treatment. Firstly, based on the different evaluation forms and index properties, the satisfaction calculation formulas of three evaluation types: clear number, interval number and language short sentence and two index properties of cost type and benefit type are defined. Then, taking the maximization of satisfaction between doctors and patients, the maximization of the number of patients receiving diagnosis and treatment and the minimization of workload difference between doctors as the decision-making objectives, the multi-objective decision-making model is constructed and solved by NSGA-II algorithm. Finally, it is proved that NSGA-II algorithm has better performance in solving the problem of doctor-patient bilateral matching by comparing the convergence, stability of solution distribution and running time of NSGA-II and NSGA-III algorithm.

At the same time, the research of this paper also has deficiencies, which need to be further deepened and improved in the future research. After using the matching method in this paper, there are still patients who have not been matched successfully. This paper does not study how to allocate these patients who have not been matched successfully. Therefore, the allocation of patients with matching failure will be further considered in future studies.

## Data availability statement

The raw data supporting the conclusions of this article will be made available by the authors, without undue reservation.

## Author contributions

JH is the main provider of the research ideas and is responsible for the control of the research process, progress, and model construction. WZ is the main writer of the paper and responsible for simulation. HG is mainly responsible for sorting out literature review and algorithm selection. All authors contributed to the article and approved the submitted version.
